# Accumulation of myeloid-derived suppressor cells (MDSCs) induced by low levels of IL-6 correlates with poor prognosis in bladder cancer

**DOI:** 10.18632/oncotarget.16386

**Published:** 2017-03-20

**Authors:** Guoliang Yang, Wenyan Shen, Yan Zhang, Mengyao Liu, Lianhua Zhang, Qiang Liu, Hui Hui Lu, Juanjie Bo

**Affiliations:** ^1^ Department of Urology, Renji Hospital, School of Medicine, Shanghai Jiao Tong University, Shanghai, China; ^2^ Department of laboratory medicine, Ren Ji Hospital, School of Medicine, Shanghai Jiao Tong University, Shanghai, China; ^3^ School of Biomedical Engineering, Shanghai Jiao Tong University, Shanghai, China; ^4^ Clinical Stem Cell Research Center, Renji Hospital, School of Medicine, Shanghai Jiao Tong University, Shanghai, China; ^5^ Department of Pathology, Renji Hospital, School of Medicine, Shanghai Jiao Tong University, Shanghai, China

**Keywords:** MDSCs, bladder cancer, IL-6, immune suppression, prognosis

## Abstract

Bladder cancer (BC) is one of the most commonly occurring cancers, with a high recurrence rate and poor outcomes in cases of relapsed metastatic disease. Here, we analyzed the markers and significance of myeloid-derived suppressor cells (MDSCs) for BC development and progression. MDSC markers were examined in peripheral blood from 113 BC patients and 20 healthy volunteers. We identified CD11b^+^CD33^low^HLA-DR^−^ CD3^−^ cells as markers of MDSCs in peripheral blood from BC patients. We also demonstrated that MDSC numbers are higher in BC patients than healthy donors, and that MDSC numbers correlate with the clinical grade, stage, and poor prognosis. In addition, serum IL-6 levels are decreased in BC patients with higher MDSC counts. IL-6 blockade increases induction of MDSCs *in vitro*. Low IL-6 levels inhibit activation of Stat3, resulting in the increased formation of MDSCs in BC. These results indicate that the MDSCs numbers may serve as a novel prognostic marker in BC patients, and that targeting IL-6 signaling may be a promising strategy for BC treatment.

## INTRODUCTION

Bladder cancer (BC) is the second most common genitourinary malignancy [1]. At initial diagnosis, 75% of BC patients present with non-muscle-invasive bladder cancer (NMIBC) and can be managed with transurethral resection (TUR) and intravesical therapy. The remaining 25% of BC patients present with muscle-invasive bladder cancer (MIBC), for which the standard treatment is radical cystectomy (RC), and which is frequently associated with metastatic disease and increased mortality. Despite improvement in surgical techniques, 5-year disease-free survival (DFS) and cancer-specific survival (CSS) rates after RC remain between 50 and 70% [2–4].

Previous studies have revealed a strong correlation between inflammation and cancer incidence [5, 6], suggesting that immune cells play an important role in cancer development and progression. Chronic inflammation contributes to tumor initiation and progression via both non-immune and immune mechanisms [7]. Most cancers are characterized by the overproduction of immunosuppressive cells and cytokines [8]. In particular, myeloid-derived suppressor cells (MDSCs) have become the focus of intense study in recent years because of their important role in tumor-associated immune suppression. MDSCs represent a heterogeneous population of cells consisting of myeloid progenitor cells and immature myeloid cells that can suppress T cell responses [9]. In mice, MDSCs are characterized by the expression of CD11b and Gr-1, while in humans, the MDSCs markers are variable, and depend on the type of malignancy. Human MDSCs are generally considered to be myeloid-derived, non-lineage determined, and with poor antigen presentation capacity. The phenotypes of MDSCs isolated from different types of cancer patients have been identified [10–13]. Increased numbers of MDSCs have been observed in various solid and hematologic malignancies, and have been associated with cancer progression and tumor-induced immune dysfunction [14–16].

Although increased accumulation of MDSCs has been reported in patients with BC [17], the specific MDSCs markers and underlying mechanisms remain unknown. Therefore, in this study, we assessed the predictive value of MDSCs in patients with BC. In addition, MDSCs are induced and/or activated by pro-inflammatory mediators [18–20]. If inflammation-induced MDSCs are an important link between inflammation and cancer, then the regulation of inflammatory mediators could affect MDSCs levels and delay tumor progression. To assess this hypothesis, we evaluated the relationship between cytokines responsible for the accumulation and activities of MDSCs and the amount of circulating MDSCs in BC patients. Our results demonstrate that decreased levels of IL-6 contribute to the accumulation of MDSCs in BC patients, suggesting novel strategies for the development of immune-based therapies.

## RESULTS

### MDSCs in BC patients are CD11b^+^CD33^low^HLA-DR^−^CD3^−^ cells

**Figure 1:**
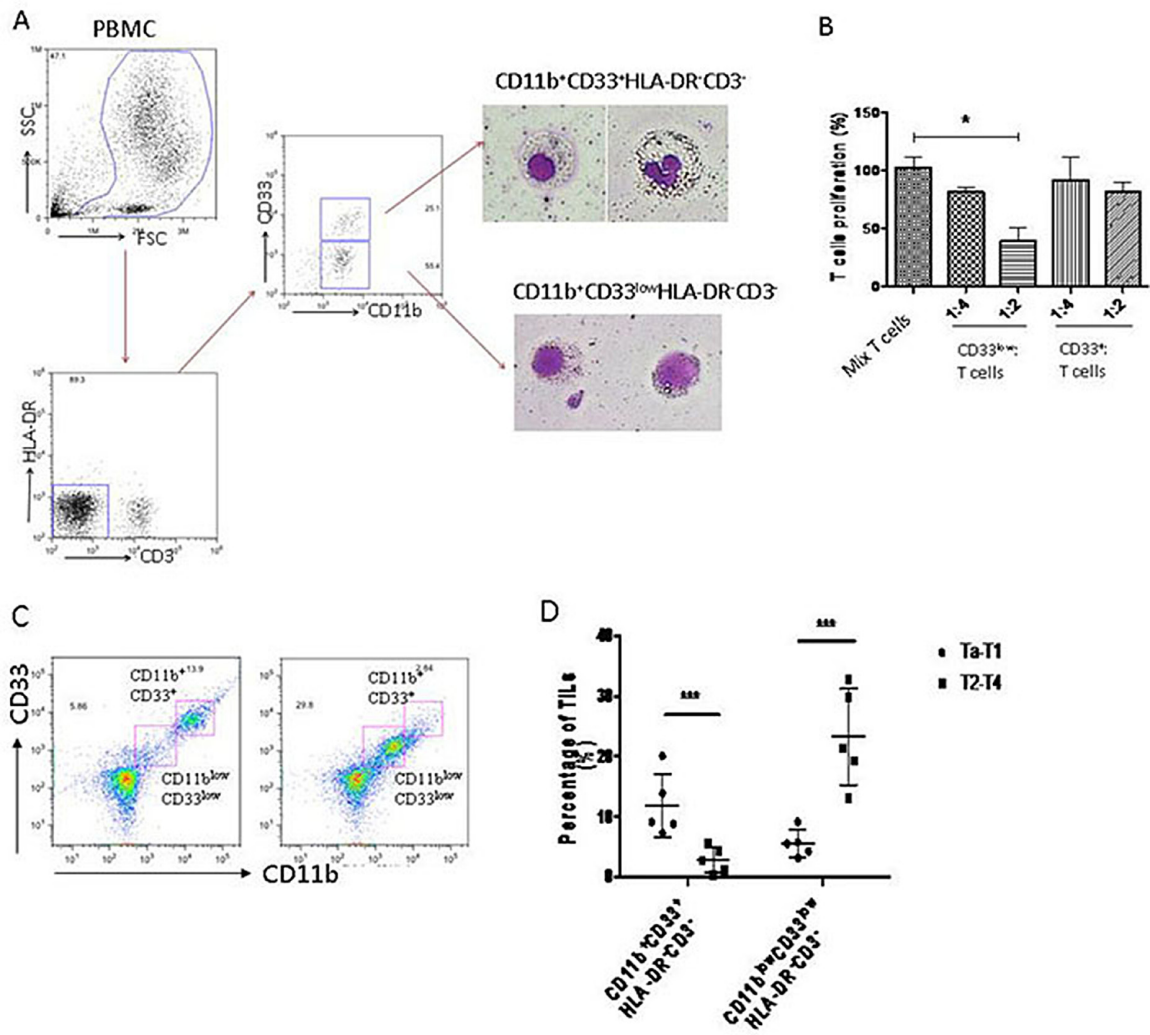
MDSCs of human bladder cancer are defined as CD11b+CD33lowHLA-DR−CD3− cells (**A**) Flow cytometry was performed on freshly isolated PBMCs from BC patients. Morphological characterization of myeloid derived suppressor cells (MDSCs) sorted and analyzed by Giemsa staining. (**B**) Circulating CD11b^+^CD33^low^HLA-DR^−^CD3^−^ cells suppress autologous T cell proliferation at a 2:1 T cell/MDSC ratio (*P < 0.05). (**C**) MDSCs (CD11b^low^ CD33^low^HLA-DR^−^CD3^−^) were accumulated in tumor tissue of BC patients. (**D**) MDSCs (CD11b^low^ CD33^low^HLA-DR^−^CD3^−^) were dramatically higher in patients at stage of Ta-T1 than those at stage of T2-T4 (***P < 0.01).

The cell surface markers for human MDSCs are variable in different types of tumors. To determine the MDSCs phenotype in BC patients, we isolated peripheral blood mononuclear cells (PBMC) from peripheral blood of BC patients, and stained them with common MDSCs markers. The results showed that there were two main groups of immature myeloid cells (CD11b^+^HLA-DR^−^CD3^−^) in PBMC of BC patients: one was CD33^low^, and the other was CD33^+^ (Figure 1A). These cells were isolated by flow-cytometry and stained by Giemsa. The morphology showed that CD33^+^ cells had segmented nuclei like mature granulocytes, while CD33^low^ cells had big and round nuclei like immature myeloid cells (Figure 1A). To confirm that the CD11b^+^CD33^low^HLA-DR^−^CD3^−^cells are MDSCs, we examined their suppressive effects on T cell proliferation. The results showed that CD33^low^ cells significantly inhibited T cell proliferation, suggesting that the CD33^low^ CD11b^+^HLA-DR^−^CD3^−^ cells are MDSCs of BC patients (Figure 1B).

To confirm that the MDSCs were present in tumor tissues of BC patients, we collected the BC tumor samples, and analyzed the tumor-infiltrated leukocytes by flow cytometry. The data showed an increased accumulation of MDSCs (CD11b^low^CD33^low^HLA-DR^−^CD3^−^) in tumor tissues of BC patients (Figure 1C). The MDSCs accumulation was significantly increased in BC patients with stage Ta-T1 compared to patients with stage T2-T4 (Figure 1D).

### MDSCs are increased in peripheral blood of BC patients at advanced stages

**Figure 2:**
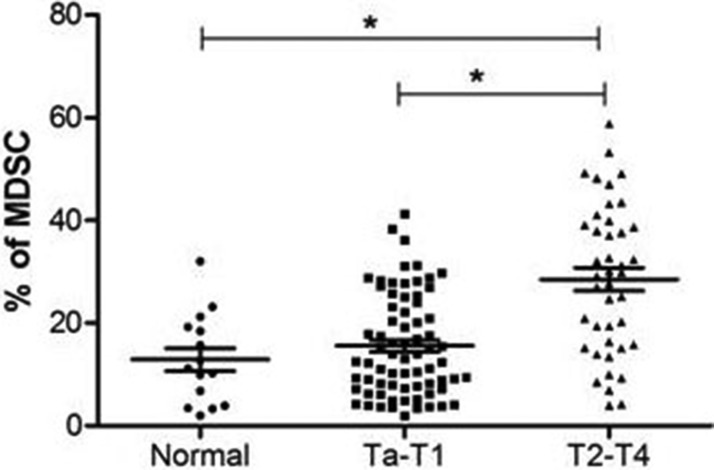
The percentage of MDSCs is increased in bladder cancer patients The proportion of MDSCs in peripheral blood from BC patients was significantly increased compared with the proportion from healthy donors, and the level of MDSC was higher in BC patients at stage of T2-T4 than that of Ta-T1 (*P < 0.05).

To evaluate the significance of MDSCs on BC prognosis, we investigated the proportion of MDSCs (CD11b^+^CD33^low^HLA-DR^−^CD3^−^) cells in BC patients (*n* = 113) and healthy donors (*n* = 20). The proportion of MDSCs in peripheral blood from BC patients was significantly increased compared with the proportion from healthy donors. In addition, the level of MDSCs was higher in BC patients at stage of T2-T4 than that of Ta-T1 (*P* < 0.05) (Figure 2).

### Correlation between MDSCs and clinicopathological features

To determine the clinical significance of MDSCs, 113 BC patients were enrolled and divided into two groups according to the frequency of MDSCs. Values that were higher than the mean of all tumors were considered elevated. For the low group, the frequency of MDSCs was defined as less than or equal to 21%, whereas for the high group, the frequency of MDSCs was greater than 21%. We found that the higher frequency of MDSCs was associated with tumor grade and stage (*P* = 0.009 and *P* < 0.001, respectively). However, the higher frequency of MDSCs was not associated with other clinicopathological features, such as age, sex, tumor size, and tumor number (Table 1).

**Table 1:** Correlations between the proportion of MDSC and clinicopathological features in 113 patients with bladder cancer

Parameter	Case	MDSC expression		*P* value
		Low	High	
Sex				0.367
Female	34	18	16	
Male	79	49	30	
Age (years)				0.602
≤ 65	41	23	18	
> 65	72	44	28	
Tumor size(cm)				0.450
≤ 3	74	42	32	
> 3	39	25	14	
Tumor number				0.127
Unifocal	64	34	30	
Multifocal	49	33	16	
Grade				0.009
low	38	29	9	
high	75	38	37	
T stage				< 0.0001
pTa- pT1	71	51	20	
pT2- pT4	42	16	26	

### Circulating MDSCs levels correlate with poor outcome in BC patients

**Figure 3:**
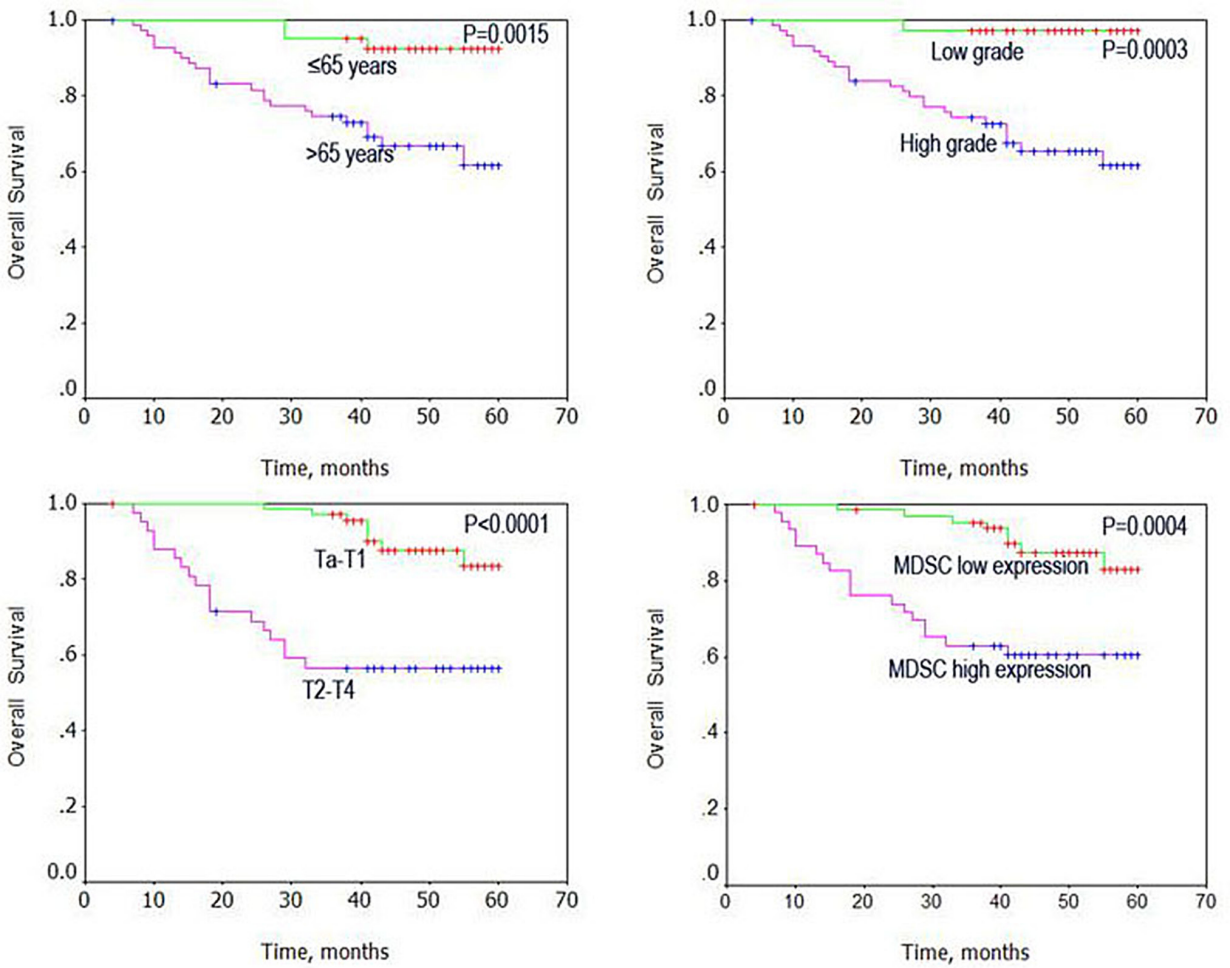
Univariate analysis of overall survival in patients with bladder cancer using the Kaplan Meier method

Using Kaplan–Meier analysis method, we observed that age, grade, stage, and high MDSCs levels correlated with OS (both *P* < 0.001, Table 2). The log-rank test further demonstrated that the overall survival time was associated with age, grade, stage, and high MDSCs levels (Figure 3). Multivariate analysis was also performed with the Cox proportional hazards model including gender, age, tumor size, number, grade, and stage, and the MDSCs levels. The results showed that the MDSCs levels correlated with BC prognosis, and were an independent prognostic factor for patients with BC (*P* = 0.006; Table 3)

**Table 2:** Univariate survival analysis of overall survival in 113 patients with BC

Variable	case	Overall survival		
		Mean ± SE(month)	95%CI	*P* value
Gender				0.415
Female	34	49 ± 3	(43–54)	
Male	79	52 ± 2	(49–56)	
Age (yeas)				0.0015
≤ 65	41	58 ± 1	(56–60)	
> 65	72	48 ± 2	(43–52)	
Tumor size (cm)				0.6294
≤ 3	74	52 ± 2	(48–56)	
> 3	39	50 ± 3	(45–55)	
Tumor number				0.5258
Unifocal	64	53 ± 2	(49–56)	
Mutlifocal	49	50 ± 3	(45–55)	
Grade				0.0003
Low	38	59 ± 1	(57–60)	
High	75	48 ± 2	(43–52)	
T stage				< 0.0001
Ta-T1	71	57 ± 1	(55–59)	
T2-T4	42	42 ± 3	(35–48)	
Proportion of MDSC				0.0004
Low	67	57 ± 1	(44–59)	
High	46	44 ± 3	(38–50)	

**Table 3:** Multivariate analyses of clinicopathological factors for the overall survival of the patients with BC

Variable	Multivariate analysis		
	Hazard ratio	95% CI	*P* value
Gender			0.610
Male vs female	0.797	0.333–1.908	
Age(years)			0.002
≤ 65 vs > 65	6.940	1.994–24.148	
Tumor number			0.954
Unifocal vs mutlifocal	1.027	0.413–2.551	
Tumor size(cm)			0.579
≤ 3 vs > 3	0.782	0.327–1.867	
Grade			0.066
Low vs high	7.243	0.878–59.746	
T stage			0.062
pTa- pT1 vs pT2- pT4	2.586	0.955–7.006	
Proportion of MDSC			0.006
Low vs high	3.582	1.448–8.861	

Abbreviations: CI = confidence interval.

### Serum levels of MDSC-inducing cytokines differ between Ta-T1 and T2-T4 stages in BC patients

Tumor cells or stroma cells in tumor environment can release soluble factors that affect the differentiation of myeloid cells [21]. Some cytokines, such as GM-CSF, G-CSF, VEGF, or IL-3, play an important role in myelopoiesis [22], while other cytokines, such as IL-1β and IL-6, contribute to the immunosuppressive activity of MDSCs [23].

**Figure 4:**
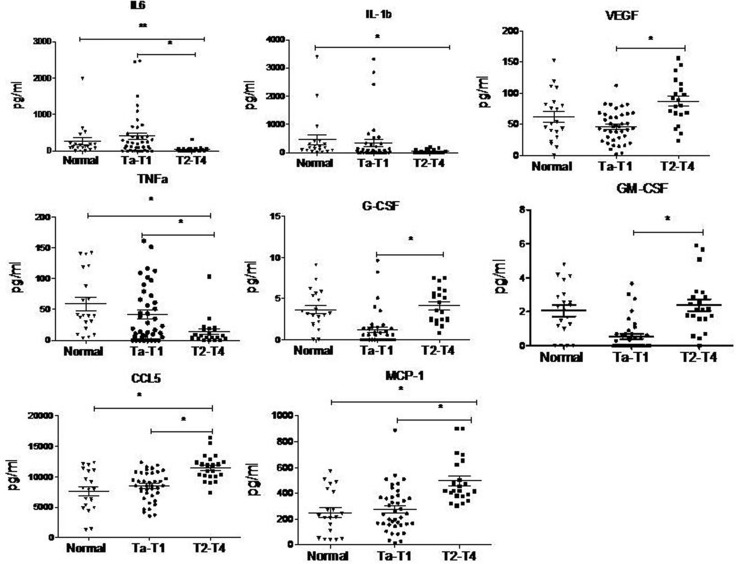
Cytokine levels in serum of BC patients analyzed by cytometric beads assay The levels of VEGF, G-CSF, GM-CSF, CCL5 and MCP-1 are up-regulated while the levels of IL-6, IL-1b and TNFα are down-regulated in BC patients with T2-T4 stage, compared to BC patients with Ta-T1 stage and healthy control. (*P < 0.05)

To explore the mechanism responsible for the formation of MDSCs in BC patients, we examined the serum levels of MDSCs-inducing cytokines in BC patients by cytometric beads assay. Serum was obtained from 20 healthy donors and 61 BC patients. The levels of VEGF, G-CSF, GM-CSF, CCL5, and MCP-1 were increased, while the levels of IL-6, IL-1β and TNFα were decreased in BC patients with T2-T4 stage, compared to BC patients with Ta-T1 stage, and healthy controls (Figure 4).

### Low level of IL-6 is sufficient to induce MDSCs formation *in vitro*

**Figure 5:**
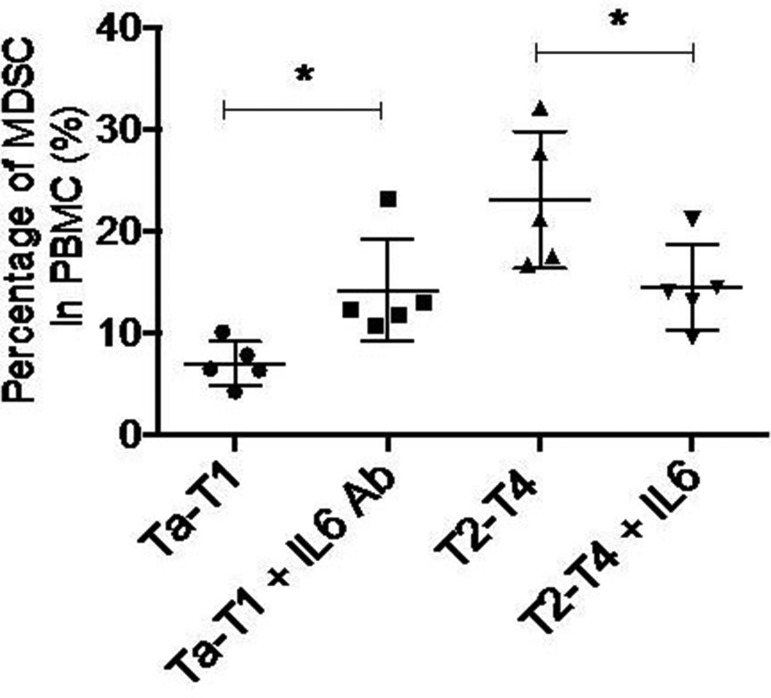
Down-regulation of IL6 is sufficient to induce MDSC formation *in vitro* Adding exogenous IL6 to serum gotten from BC patients in T2-T4 stage significantly undermined the accumulation of MDSC, while adding IL6 neutralizing antibody (5E1) to serum of BC patients in Ta-T1 stage increased the number of MDSC(*P < 0.05).

Among the eight cytokines examined by cytometric beads assay, only the levels of IL-6, TNFα, CCL5, and MCP-1 were significantly changed in both Ta-T1/T2-T4 and control/T2-T4 BC patients. To determine which specific cytokine contributes to the accumulation of MDSCs in stages T2-T4, we induced MDSCs formation *in vitro* by adding individual cytokines or corresponding neutralizing antibodies. The results showed that adding exogenous IL-6 to serum obtained from BC patients in T2-T4 stage significantly decreased the accumulation of MDSCs, while adding IL-6 neutralizing antibody (5E1) to serum of BC patients in Ta-T1 stage increased the number of MDSCs (Figure 5). Modulation of TNFα, CCL5, or MCP-1 levels had no effect on MDSCs accumulation (data not shown). These data suggest that low levels of IL-6 induce the differentiation of MDSCs cells.

### Stat3, but not NF-kB, regulates IL-6-mediated differentiation of MDSCs

**Figure 6:**
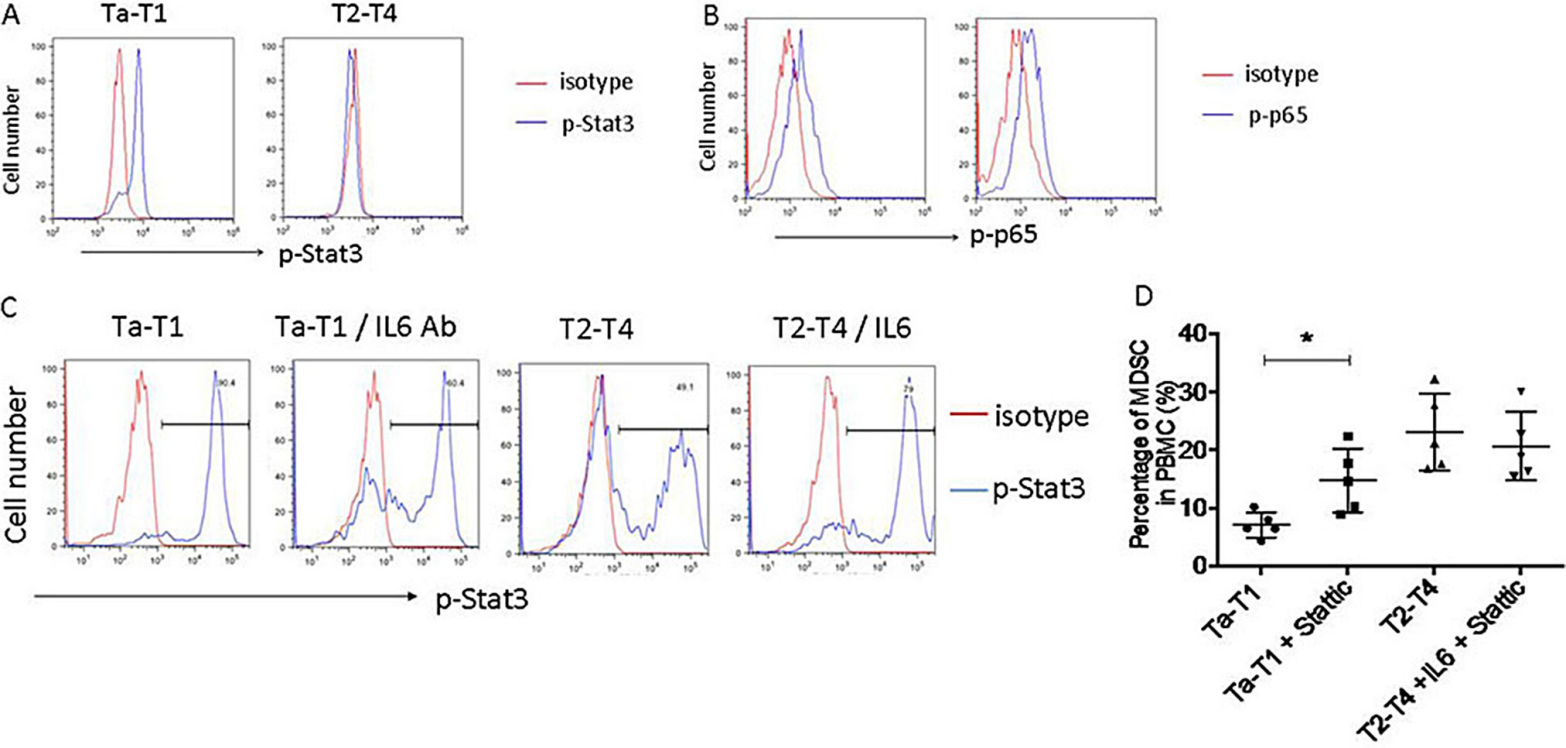
Stat3, but not NF-kB, regulates differentiation of MDSC induced by low levels of IL-6 (**A** and **B**) The phosphorylation of Stat3, not that of p65, is dramatically down-regulated in patients in T2-T4, compared to those in Ta-T1. (**C**) In the experiment of induction of MDSC *in vitro*, the phosphorylation of Stat3 was dramatically inhibited by serum of BC patients at stage of T2– T4, and the level of p-Stat3 was regulated by adding IL6 protein or IL6 neutralizing antibody. (**D**) The effect of Stattic on MDSC formation induced by serums of BC patients *in vitro*.

To investigate the signaling pathways involved in the IL-6-mediated MDSCs differentiation, we examined the IL-6-induced activation of NF-κB (phosphorylation of p65) and Stat3 (phosphorylation of Stat3) pathways in CD11b^+^ myeloid cells in BC patients in T2-T4 and Ta-T1 stages. Phosphorylation of Stat3, but not p65, was decreased in BC patients in T2-T4 stages, compared to Ta-T1 stages (Figure 6A and 6B). To confirm the role of IL-6/Stat3 in the differentiation of MDSCs, we examined the levels of p-Stat3 in MDSCs induced by serum of BC patients. Figure 6C shows that Stat3 phosphorylation was decreased by adding serum from BC patients at stages T2-T4, and that it was regulated by adding IL-6 protein or IL-6 neutralizing antibody.

To examine the effect of IL-6/STAT3 signaling on MDSCc formation, we used the STAT3 inhibitor, Stattic (10 μM). In the presence of serum from Ta-T1 BC patients, Stattic significantly increased the percentage of MDSCs compared to the control group. In the presence of serum from T2-T4 BC patients, Stattic blocked the IL6-induced inhibition of MDSCs (Figure 6D). Together, our data indicate that the IL-6/Stat3 signaling plays an important role in the differentiation of MDSCs.

## DISCUSSION

The immune system plays a pivotal role in the development, progression, and metastasis of human BC. Although the precise mechanisms remain elusive, clinical studies have indicated that MDSCs promote tumor progression and present an important barrier limiting the full potential of immune-based cancer therapies [24]. Additionally, MDSCs have been associated with poor prognosis in several types of cancer [7, 8, 25].

In the present study, we have identified the markers for MDSCs in Chinese BC patients and characterized the percentage of CD33^low^CD11b^+^HLA-DR^−^CD3^−^myeloid cells in BC patients. The data revealed that the number of CD33^−^CD11b^+^HLA-DR^−^ CD3^−^cells significantly increased in the PBMC of BC patients compared with healthy donors. Since these cells exerted potent immunosuppressive activity on T cells, we have defined them as MDSCs in bladder cancer. Moreover, the proportion of MDSCs cells correlated with BC tumor grade and stage, and could predict a poor outcome.

LIN^−^HLA^−^DR^−^CD33^+^CD11b^+^ MDSCs have been identified in patients of glioblastoma, breast cancer, colon cancer, lung cancer, and kidney cancer [14, 15, 26–28]. Previous studies have shown that the number of these myeloid cells may reflect the clinical stages of tumors, and positively correlate with poor prognosis in patients with breast and colorectal cancer [14–16]. It is important that the phenotype of MDSCs appears to be influenced by the type of cancer [21], such as in renal cancer, CD11b^+^CD14^−^CD15^+^CD66b^+^VEGFR1^+^ cells have immunosuppressive activity [29]. A recent study performed by USA researchers has shown that innate granulocytes with phenotypes of CD15^hi^CD33^low^ in blood, and CD11b^+^CD15^+^HLA-DR^−^ in tumor tissues, are increased and activated in BC patients, and that these cells exert immunosuppressive activity on T cell proliferation *in vitro* [17]. However, in Chinese BC patients, we did not observe a significant difference in CD15 expression in myeloid cells, and we identified CD11b^+^CD33^low^HLA-DR^−^ CD3^−^ as the markers for MDSCs, which are significantly increased in BC patients at stages of T2-T4.

MDSCs are recruited by multiple pro-inflammatory molecules secreted by cancer or stroma cells to the tumor sites where they exploit a plethora of redundant mechanisms to inhibit immune responses, including depleting required nutrients for lymphocytes, generating oxidative stress, interfering with lymphocyte trafficking and viability, and activating and expanding the T-reg population [21, 28]. Recent evidence suggests that MDSCs accumulation is induced by numerous cytokines and soluble mediators, including macrophage colony-stimulating factor (M-CSF), G-CSF, granulocyte-macrophage colony-stimulating factor (GM-CSF), IL-6, IL-1, TNF, and S100A8/S100A9 [30–36]. Our study shows that IL-6 levels are decreased in BC patients compared with healthy donors. Moreover, there is a significant correlation between plasma IL-6 levels and frequency of MDSCs.

IL-6 is a multifunctional cytokine that plays an important role in a wide range of biologic activities in different cell types, including inflammatory and tumor cells. Our study shows that the decreased levels of IL-6 in PBMC promote the formation of MDSCs *in vitro*, and enhance the immunosuppressive functions of PBMC. Stat3 is a transcription factor important for myeloid lineage development and differentiation. Activation of Stat3 increases macrophage defects and MDSCs expansion [37]. Stat3 signaling in MDSCs may also be potentially modulated by IL-6 [23, 38].

An important finding in this study is that IL-6 inhibits the accumulation of MDSCs via the activation of Stat3, but not NF-κB. Activation of Stat3 and NF-κB regulates the expression of apoptotic, proliferative and immune response genes [35, 39–41]. Inhibition of NF-κB and Stat3 activation has become an effective therapeutic anti-cancer strategy [41–43]. We found that the activation of Stat3, but not NF-κB, is inhibited in MDSCs, because of the decreased levels of IL-6.

In summary, our study indicates that the levels of MDSCs may predict the prognosis of BC patients. Moreover, decreased expression of IL-6 increases MDSCs accumulation, thus providing the immunosuppressive microenvironment for the development of bladder cancer. Therefore, targeting the IL-6 signaling may be a promising strategy for the treatment of bladder cancer.

## MATERIALS AND METHODS

### Patients

Blood samples from BC patients and healthy donors were collected from our hospital between January 2010 and December 2011. The criteria for study enrollment were histopathological diagnosis of transitional cell carcinoma of the bladder, newly diagnosed and untreated, no history of other tumor, and the potential for follow up. We excluded carcinoma *in situ* from our study. Prior patient's consent was obtained from all patients and healthy donors. The study was approved by the Ethics Committee of our hospital. Clinical information about the samples is described in detail in Table 1. The patients included 79 males and 34 females, ranging between 45 and 84 years (mean age, 66.5 years). The median follow-up time for overall survival (OS) was 43 months at the time of analysis, and ranged from 4 to 60 months. OS was defined as the interval between BC resection and death; patients alive at the end of follow-up were monitored.

### Cell isolation and staining

MDSCs were isolated from PBMC using anti-CD33 magnetic microbeads (Miltenyi Biotec), following manufacturer's instruction. Cells were treated with 10 μg/ml anti-GM-CSF antibody (Ab) and 15 μg/ml anti-CCL5 Ab (R&D system). The immunosuppressive activity of MDSCs was evaluated by their ability to inhibit the proliferation of autologous T cells. T cells were purified from PBMC of autologous donors using CD3+ T-cell enrichment column (R&D Systems), following manufacturer's instruction. T cells were added to MDSCs in a 2:1 ratio, and stimulated by anti-CD3/CD28 stimulation beads (Invitrogen) and IL-2 (100 u/ml). Two days later, 10 mM ErdU was added to the culture and cells were allowed to grow for another 24 h before being harvested and analyzed by flow cytometry.

### Cytometric bead arrays

Serum levels of IL-6, IL-1β VEGF, TNFα, G-CSF, GM-CSF, CCL5, and MCP-1 were analyzed using the human CBA flex set (BD Pharmingen, USA).

### Induction of MDSC *in vitro*

PBMC from healthy donors were isolated by differential density gradient separation. PBMC were cultured in complete medium with GM-CSF (10 ng/ml) and IL-6 (10 ng/ml) for 7 days. MDSCs were analyzed by flow cytometry, and characterized by the CD11b+CD33lowHLA-DR- CD3- phenotype.

### Statistical analysis

Differences between healthy controls and bladder cancer patients were evaluated by the Mann–Whitney *U* test. The significance of the relationships between MDSCs and clinicopathological parameters was evaluated using chi-squared tests. OS curves were calculated using the Kaplan–Meier method and compared by log-rank test. The significance of various variables for OS was analyzed by the Cox proportional hazards model in the multivariate analysis. SPSS 11.0 software (SPSS, Inc., Chicago, IL) was used for statistical analysis. *P* values < 0 .05 were considered statistically significant. Figures were generated in GraphPad Prism (version 5.0, GraphPad Software).
